# The *Encarsia flavoscutellum*-group key to world species including two new species from China (Hymenoptera, Aphelinidae)

**DOI:** 10.3897/zookeys.662.11809

**Published:** 2017-03-21

**Authors:** Hui Geng, Cheng-De Li

**Affiliations:** 1 School of Forestry, Northeast Forestry University, Harbin, 150040, China

**Keywords:** Chalcidoidea, China, *Encarsia baoshana*, *Encarsia longchuana*, taxonomy

## Abstract

Two new species of *Encarsia flavoscutellum*-group, *E. baoshana* Li & Geng, **sp. n.** and *E. longchuana* Li & Geng, **sp. n.** are described from China, and photomicrographs are provided to illustrate morphological characters of the new species. A key to all six described species of the *E. flavoscutellum*-group is given.

## Introduction

*Encarsia* Förster is the largest genus of the family Aphelinidae, and currently contains 441 valid species worldwide, including 103 species from China, of which 45 species are endemic to China including 12 species from Taiwan (Noyes 2017; Geng and Li 2013, 2016). Most species of *Encarsia* with known biology are primary endoparasitoids of Aleyrodidae or Diaspididae (Hemiptera).

The *Encarsia flavoscutellum* species group was established by Evans et al. (1995) including four species, *E. thoracaphis* (Ishii, 1938), *E. noordami* Polaszek, 1995, *E. cerataphivora* Evans, 1995 and *E. flavoscutellum* Zehntner, 1900 which are known to be specifically parasitoids of Hormaphidinae (Hemiptera, Aphididae). Members of the *flavoscutellum*-group can be recognized by the following combination of characters:

*Females*: Scutellum always pale. Third valvulae dark in contrast to the second valvifers. Club 3-segmented. Mandibles with two teeth and a truncation. Mid lobe of mesoscutum with 4–6 pairs of setae. Each side lobe with 3 setae. Axilla large and long, each with a single robust seta centrally, towards the inner margin of the axilla. Scutellum large, as broad as, and more than half as long as, mid lobe of mesoscutum. Scutellar sensilla widely separated. Sculpture on dorsum of mesothorax reticulate and generally robust, scutellum centrally with elongate cells, as in most *Encarsia*. Tarsal formula 5-5-5. Fore wing uniformly setose.

*Males*: As females except for genital characters and the following: antenna with F2 ventrally bearing a distinctive sensorial complex, F5 partially or completely fused with F6.

Here two new species of this group are described from China. A tentative key to all the known species is provided based on their original descriptions.

## Material and methods

Specimens were collected from Yunnan, Henan, Shaanxi and Sichuan Provinces, China by sweeping or using yellow pan traps. Specimens were dissected and mounted dorsally in Canada balsam on slides following the method of Noyes (1982) and morphological terminology following Huang & Polaszek (1998) except metasoma is used for the petiole plus gaster.

Photographs were taken with a digital CCD camera attached to an Olympus BX51 compound microscope, and most measurements were made from slide-mounted specimens using an eye-piece graticule. All the specimens listed below are deposited in Northeast Forestry University, Harbin, China.

The following abbreviations are used:

**OOL** minimum distance between a posterior ocellus and the corresponding eye margin;

**POL** minimum distance between posterior ocelli;

**Fn** flagellar segment;

**Tn** gastral tergum;

**YPT** yellow pan trapping.

Abbreviations for depositories:

**NEFU** Northeast Forestry University, Harbin, China.

**USNM** United States National Museum of Natural History, Washington DC, USA.

### Key to species of *Encarsia flavoscutellum*-group (females)

**Table:** 

1	Length of second valvifer and third valvula combined distinctly longer than hind tibia. Mandibles weakly dentate	**2**
–	Length of second valvifer and third valvula combined as long as, or distinctly shorter than hind tibia. Mandibles strongly dentate.	**4**
2	Legs with all coxae and femora dark brown; fore wing with a very slight infuscation below marginal vein; mandible teeth rather blunt, with only a single small apical tooth and a broad truncation; F1 with 4–5 longitudinal sensilla	***E. thoracaphis* (Ishii)**
–	Legs entirely yellow, or at most hind coxae brownish; fore wing hyaline; mandibles with two week teeth and a truncation; F1 with 2–3 longitudinal sensilla	**3**
3	Occiput entirely brown; mid lobe of mesoscutum largely and axillae brown; hind wing relatively narrow and disc sparsely setose; F1 0.8–0.83× as long as pedicel; ovipositor 1.07–1.15× as long as mid tibia, third valvula 0.41–0.43× as long as second valvifer	***E. baoshana* Li & Geng, sp. n.**
–	Occiput above occipital foramen brown; mid lobe of mesoscutum largely yellow except anterior half centrally brown, axillae yellow; hind wing relatively wider and disc densely setose; F1 as long as pedicel; ovipositor 1.24× as long as mid tibia, third valvula 0.36× as long as second valvifer	***E. longchuana* Li & Geng, sp. n.**
4	F1 without longitudinal sensillum; metasoma pale brown and thoracic setae pale	***E. noordami* Polaszek**
–	F1 with 1–3 longitudinal sensilla; metasoma dark brown and thoracic setae dark	**5**
5	F1 clearly shorter than both pedicel and F2; F2 and F3 slightly longer than wide. [Male with F5 and F6 partialy fused]	***E. cerataphivora* Evans**
–	F1 as long as pedicel or F2; F2 and F3 twice as long as wide. [Male with F5 and F6 completely fused]	***E. flavoscutellum* Zehntner**

## Taxonomy

### Encarsia baoshana

Taxon classificationAnimaliaHymenopteraAphelinidae

Li & Geng sp. n.

http://zoobank.org/5CF0C496-B829-4EE0-A8B8-1931071D0D11

Figs 1–7 , 8–13

#### Type material.

Holotype. ♀ [on slide, NEFU], CHINA, Yunnan Province, Baoshan City, Taibao Park, 4. V. 2013, Xiang-Xiang Jin, Guo-Hao Zu, Chao Zhang, ex from an unidentified aphid.

Paratypes. 2♀, 1♂ [on slides, NEFU], same data as holotype.

#### Diagnosis.

*Female.* Length, mesosoma plus metasoma, 0.63–0.71mm. Head with occiput dark brown. Mid lobe of mesoscutum mostly dark brown. Wings hyaline. Legs pale yellow with hind coxae pale brown. Metasoma dark brown except apex of T7 pale yellow. Frontovertex with transverse rugose sculpture. Mandibles weakly dentate, with two weak teeth and a truncation. F1 shorter than F2 and F3 respectively, with 1–2 longitudinal sensilla. Ovipositor 1.07–1.15× as long as mid tibia, and 0.84–0.89× as long as mid tibia and basitarsus combined. Length of second valvifer and third valvula combined 1.28–1.35× as long as hind tibia.

*Male*. Ocellar area brown, side lobes of mesoscutum entirely brown, mid coxae, hind coxae and femora brown. F5 and F6 partially fused. Genitalia 0.7× as long as mid tibia.

#### Description.

*Female.* Holotype. Length, mesosoma plus metasoma, 0.71mm. Head yellowish brown, occiput, clypeus, malar sulcus, two postocellar bars and a large patch under each eye dark brown. Eyes dark red, ocelli red. Antennae pale brown. Mesosoma with posterior part of mid lobe and scutellum yellow, expanded part of side lobe with a brown patch, metanotum and propodeum yellow. Wings hyaline, venation pale brown. Legs pale yellow except last tarsi and hind coxae pale brown. Metasoma dark brown except apex of T7 pale yellow. Ovipositor with second valvifer brown, third valvula dark brown to blackish brown.

**Figures 1–7.**
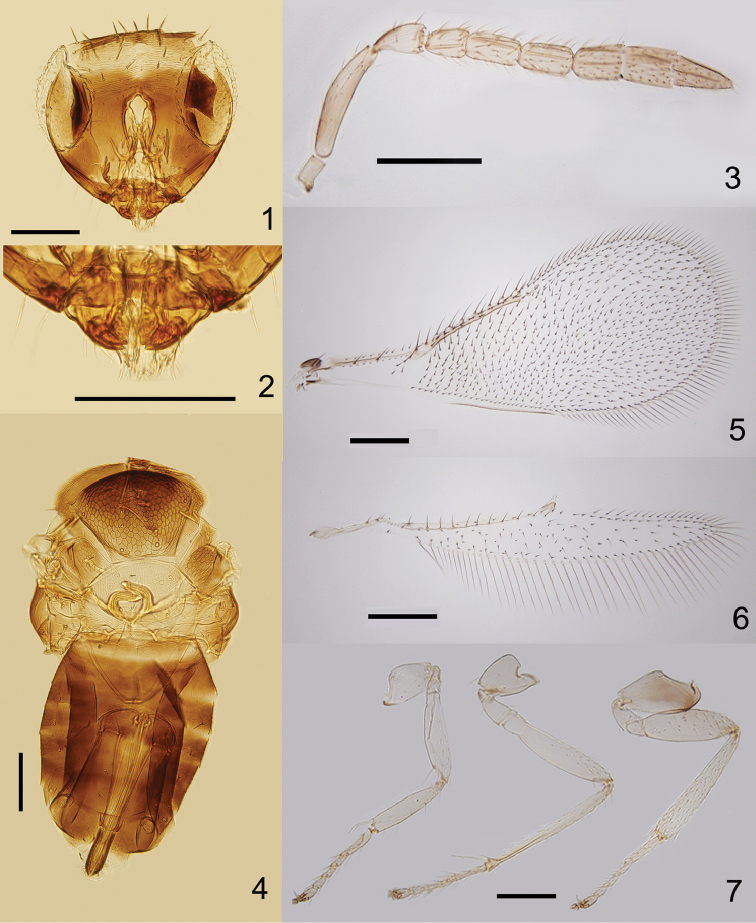
*Encarsia baoshana* sp. n., holotype ♀: **1** head, frontal view **2** mandibles **3** antenna **4** mesosoma and metasoma **5** fore wing **6** hind wing **7** legs (the same scale as Fig. 4). Scale bars 100 μm.

Head (Fig. 1) wider than mesosoma. Maxillary and labial palps 1-segmented. Mandibles (Fig. 2) with two weak teeth and a truncation. Eyes with fine and transparent setae. Frontovertex with robust setae. Antennal formula 1,1,3,3 (Fig. 3); F1 1.26× as long as wide, 0.83× as long as pedicel, clearly shorter than F2 and F3 respectively. F2 twice as long as wide, approximately equal to F3. Flagellum with the following numbers of longitudinal sensilla: F1:2, F2:2, F3:3, F4:4, F5:5, F6:4.

Mid lobe of mesoscutum (Fig. 4) with 10 setae, each side lobe with 3 setae. Axilla with 1 robust seta centrally, towards the inner margin of the axilla. Mid lobe of mesoscutum, axillae and scutellum with reticulate sculpture. Scutellum 1.65× as wide as long, 0.75× as long as mid lobe of mesoscutum. Distance between placoid sensilla on scutellum 6.67× the maximum width of a sensillum. Distance between anterior pair of scutellar setae 1.10× as long as the distance between posterior pair. Endophragma long and rounded at apex, extending to the middle of T2. Fore wing (Fig. 5) 2.55× as long as wide, uniformly and densely setose except basal area below submarginal vein, marginal fringe 0.21× as long as width of disc, costal cell with 9 setae in a row, basal cell with four setae, submarginal vein with two setae, marginal vein with seven setae along anterior margin and 1.3× as long as submarginal vein. Hind wing (Fig. 6) 6.86× as long as wide, marginal fringe 1.07× as long as width of disc. Tarsal formula 5-5-5 (Fig. 7). Mid tibial spur 0.88× as long as corresponding basitarsus, the latter 0.29× as long as mid tibia. Hind tibia 0.88× as long as mid tibia.

Petiole smooth. T1–T4 laterally and T7 apically with scale like reticulation. T2–T7 with 1+1, 1+1, 1+1, 2+2, 1+4+1 and 4 setae, respectively. T7 1.46× as wide as long. Ovipositor exerted, apparently originating from posterior margin of T2, 1.12× as long as mid tibia, 0.87× as long as mid tibia and basitarsus combined. Third valvula 0.41× as long as second valvifer. Length of second valvifer and third valvula combined 1.32× as long as hind tibia. Third valvula 0.3× as long as ovipositor.

**Figures 8–13.**
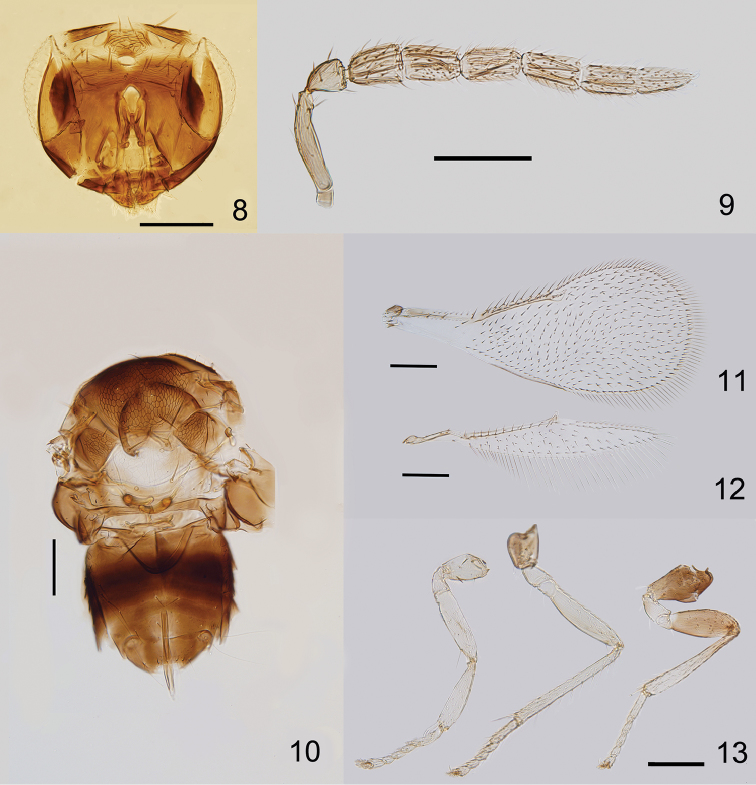
*Encarsia baoshana* sp. n., paratype ♂: **8** head, frontal view **9** antenna **10** mesosoma and metasoma **11** fore wing **12** hind wing **13** legs (the same scale as Fig. 10). Scale bars 100 μm.

*Male*. Length, mesosoma plus metasoma, 0.47mm. Head (Fig. 8) and body generally brown as in female, except ocellar area brown, side lobes entirely brown, mid coxae, hind coxae and femora brown (Fig. 13). Morphology as for female, except the following: F5 and F6 partially fused (Fig. 9). F2 with an extensive sensorial complex. Basal cell of fore wing with 2 setae (Fig. 11). Genitalia (Fig. 10) 0.7× as long as mid tibia.

#### Host.

An unidentified aphid (Hemiptera, Aphididae, Hormaphidinae).

#### Variation.

*Female*. Length, mesosoma plus metasoma, 0.63–0.71mm. Mid lobe of mesoscutum with 10–11 setae. Mid tibial spur 0.81–0.88× as long as corresponding basitarsus. Basal cell with 3–4 setae, marginal vein with 6–8 setae along anterior margin.

#### Etymology.

The specific name is derived from the collection locality name.

#### Discussion.

*Encarsia baoshana* sp. n. is close to *E. thoracaphis* (Ishii) in having a relatively longer ovipositor compared with hind tibia, and weakly dentate mandibles, but can be distinguished from the latter by the colour of legs, hyaline wings, mandibles, and number of longitudinal sensilla on F1 as listed in foregoing key. Furthermore, the length of second valvifer and third valvula combined 1.28–1.35× as long as hind tibia (*vs* 1.5×), and anterior margin of marginal vein with 6–8 setae (*vs* 10–11).

The new species is also closely related to *E. longchuana* sp. n., and the differences were listed in the key, and also see the discussion under *E. longchuana* sp. n..

### Encarsia longchuana

Taxon classificationAnimaliaHymenopteraAphelinidae

Li & Geng sp. n.

http://zoobank.org/E33CD0DD-02B1-454A-8760-6ED93E9E0574

Figs 14–21

#### Type material.

Holotype. ♀ [on slide, NEFU], CHINA, Yunnan Province, Longchuan County, 27. IV. 2013, Xiang-Xiang Jin, Guo-Hao Zu, Chao Zhang, YPT.

#### Diagnosis.

*Female.* Length, mesosoma plus metasoma, 0.77mm. Head with occiput above occipital foramen dark brown. Anterior half of mid lobe brown. Wings hyaline. Legs pale yellow. Metasoma dark brown except apex of T7 pale yellow. Mandibles with two weak teeth and a truncation. F1 slightly shorter than F2 and F3 respectively, with three longitudinal sensilla. Ovipositor 0.95× as long as mid tibia and basitarsus combined. Length of second valvifer and third valvula combined 1.4× as long as hind tibia.

#### Description.

*Female.* Holotype. Length, mesosoma plus metasoma, 0.77mm. Head yellow except occiput above occipital foramen, two postocellar bars and a large patch under each eye dark brown, clypeus and malar sulcus yellowish brown. Eyes dark red, ocelli red. Antennae yellowish brown. Mesosoma yellow, with anterior half of mid lobe and a patch on expanded part of side lobe brown. Fore wings hyaline, venation pale brown. Legs mostly pale yellow except small patches on knees and extreme apex of coxae pale brown. Metasoma dark brown except apex of T7 pale yellow. Third valvulae dark brown to blackish brown.

**Figures 14–21.**
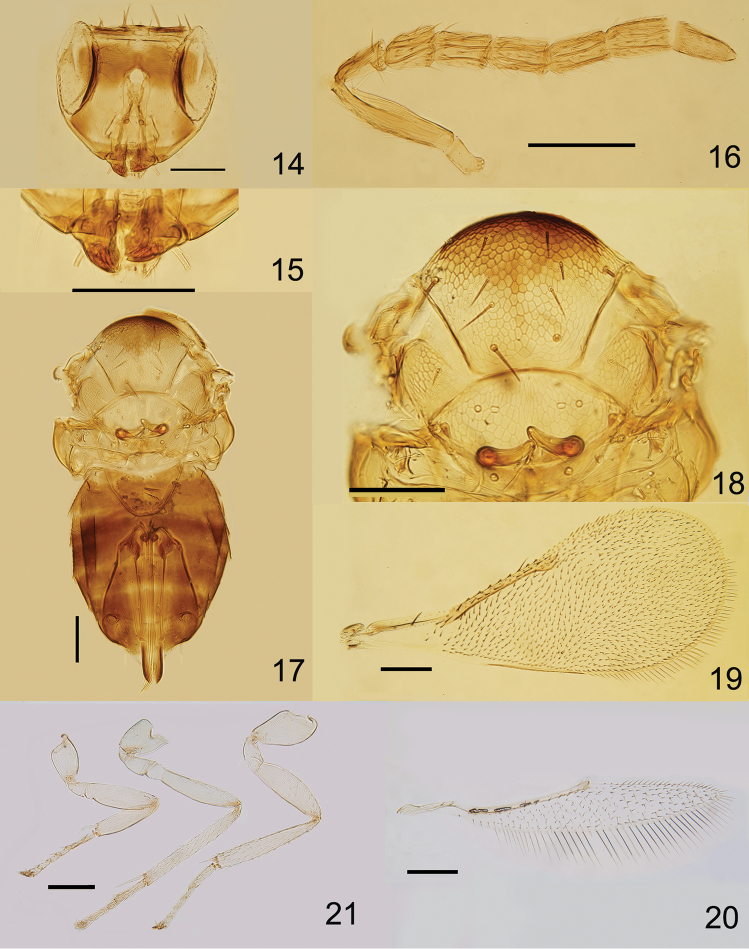
*Encarsia longchuana* sp. n., holotype ♀: **14** head, frontal view **15** mandibles **16** antenna **17** mesosoma and metasoma **18** mesosoma **19** fore wing **20** hind wing **21** legs (the same scale as Fig. 17). Scale bars 100 μm.

Head (Fig. 14). Maxillary and labial palps 1-segmented. Mandibles (Fig. 15) with two weak teeth and a truncation. POL approximately equal to OOL. Ocelli forming about an obtuse triangle. Eyes with fine and transparent setae. Frontovertex with robust setae. Antennal (Fig. 16) formula 1, 1, 3, 3; F1 1.31× as long as wide, about as long as pedicel, and 0.86× as long as F2. F2 1.69× as long as wide, approximately equal to F3. Flagellum with the following numbers of longitudinal sensilla: F1:3, F2:4, F3:4, F4:4, F5:4, F6:3.

Mesosoma 0.77× as long as metasoma (Fig. 17). Mid lobe (Fig. 18) of mesoscutum with 10 setae, each side lobe of mesoscutum with 3 setae. Axilla with 1 robust seta centrally, towards the inner margin of the axilla. Mid lobe of mesoscutum, axillae and scutellum with reticulate sculpture. Scutellum 1.47× as wide as long, and 0.87× as long as mid lobe of mesoscutum. Distance between placoid sensilla on scutellum 6.75× the maximum width of a sensillum. Distance between anterior pair of scutellar setae 1.11× as long as the distance between posterior pair. Endophragma long and rounded at apex, extending to the anterior margin of T2. Fore wing (Fig. 19) 2.44× as long as wide, uniformly and densely setose except basal area below submarginal vein, marginal fringe 0.16× as long as width of disc, costal cell with 12 setae in a row, basal cell with five setae, submarginal vein with two setae, marginal vein with eight setae along anterior margin and 1.14× as long as submarginal vein. Hind wing (Fig. 20) 6.3× as long as wide, marginal fringe 0.92× as long as width of disc. Tarsal formula 5-5-5 (Fig. 21). Mid tibial spur as long as corresponding basitarsus, and the latter 0.31× as long as mid tibia. Hind tibia 0.9× as long as mid tibia.

Petiole smooth. T1–T4 laterally and T7 apically with scale like reticulation. T2–T7 with 1+1, 1+1, 1+1, 2+2, 1+4+1 and 4 setae, respectively. T7 1.46× as wide as long. Ovipositor exerted, apparently originating from posterior margin of T2, 1.24× as long as mid tibia, and 0.95× as long as mid tibia and basitarsus combined. Third valvula 0.36× as long as second valvifer. Length of second valvifer and third valvula combined 1.4× as long as hind tibia. Third valvula 0.27× as long as ovipositor.

*Male*. Unknown.

#### Host.

Unknown.

#### Etymology.

The specific name is derived from the collection locality name.

#### Discussion.

*Encarsia longchuana* sp. n. is closely related to *E. baoshana* sp. n. in having relatively longer ovipositor compared with hind tibia, similar structure of mandibles, similar coloration of legs and wings, but can be separated from the latter by the coloration of occiput and thorax, setation of hind wing, relative length of hind wing, F1, ovipositor and the third valvula as listed in the key. Furthermore, *E. longchuana* sp. n. with clypeus and malar sulcus yellowish brown (*vs* dark brown in *E. baoshana* sp. n.) and maximum width of outer plate of ovipositor about 1.48× as wide as minimum width (*vs* 1–1.27× in *E. baoshana* sp. n.).

### Encarsia flavoscutellum

Taxon classificationAnimaliaHymenopteraAphelinidae

Zehntner, 1900

Encarsia flavo-scutellum Zehntner, 1900: 12. Neotype ♀ (USNM), Indonesia: Java, Pasoeroean, designated by Evans, Polaszek & Bennett, 1995: 34, not examined.
Encarsia flavoscutellum : Ishida, 1926: 379; Sonan, 1944: 32; Evans, Polaszek & Bennett, 1995: 34; Huang & Polaszek, 1998: 1877.

#### Material examined.

CHINA: 5♀, 2♂ [on slides, NEFU], Sichuan Province, Guangyuan City, Qingchuan County, 21. VIII. 2015, Ye Chen, Chao Zhang, sweeping; 2♀ [on slides, NEFU], Shaanxi Province, Jiange County, 18. VIII. 2015, Ye Chen, Chao Zhang, sweeping; 1♀, 1♂ [on slides, NEFU], Henan Province, Xinyang City, Shihe District, Wusheling, 7. VIII. 2015, Hui Geng, Yan Gao, Zhi-Guang Wu, sweeping.

#### Hosts.

*Ceratovacuna lanigera* Zehntner, *Astegopteryx nipae* (van der Goot) [Hemiptera, Aphididae, Hormaphidinae].

#### Distribution.

China (Shaanxi, Sichuan, Henan [new records], Guangdong, Fujian, Taiwan), India, Indonesia.

#### Discussion.

Our specimens agree with the descriptions given by Huang & Polaszek (1998), except the specimens from Sichuan and Shaanxi which have the hind coxae slightly brownish.

## Supplementary Material

XML Treatment for Encarsia baoshana

XML Treatment for Encarsia longchuana

XML Treatment for Encarsia flavoscutellum
